# Whole lesion histogram analysis of apparent diffusion coefficients on MRI predicts disease-free survival in locally advanced squamous cell cervical cancer after radical chemo-radiotherapy

**DOI:** 10.1186/s12885-019-6344-3

**Published:** 2019-11-15

**Authors:** Bo Zhao, Kun Cao, Xiao-Ting Li, Hai-Tao Zhu, Ying-Shi Sun

**Affiliations:** Department of Radiology, Key Laboratory of Carcinogenesis and Translational Research, Ministry of Education, Peking University Cancer Hospital and Institute, No. 52 Fucheng Rd, Haidian District, Beijing, 100142 China

**Keywords:** Uterine cervical neoplasm, Diffusion-weighted MRI, Survival

## Abstract

**Background:**

The aim was to investigate the prognostic value of MR apparent diffusion coefficients (ADC) using histogram analysis (HA) in predicting disease-free survival (DFS) of cervical cancer after chemo-radiation therapy.

**Methods:**

We retrospectively analyzed 103 women with pathologically proven squamous cell uterine cancer who received chemo-radiation therapy between 2009 and 2013. All patients were followed up for more than 2 years. Pre-treatment MR images were retrieved and imported for HA using an in-house developed software program based on 3D Slicer. Regions of interest of whole tumors were drawn manually on DWI with reference to T2WI. HA features (mean, max, min, 50, 10, 90%, kurtosis, and skewness) were extracted from apparent diffusion coefficient (ADC) maps and compared between the recurrence and non-recurrence groups after the 2-year follow-up. Univariate and multivariate Cox regression analysis was used to correlate ADC HA features and relevant clinical variables (age, grade, maximal diameter of tumor, FIGO stage, SCC-Ag) with DFS.

**Results:**

One hundred three patients with stage IB-IV cervical cancers were followed up for 2.0–94.6 months (median 48.9 months). Twenty patients developed recurrence within 2 years. In the recurrence group, the min (*P* = 0.001) and 10% (*P* = 0.048) ADC values were significantly lower than those of the non-recurrence group. Univariate and multivariate Cox regression analysis revealed that ADC_min_ (*P* = 0.006, HR = 0.110) was significantly correlated with DFS.

**Conclusion:**

Pre-treatment volumetric ADC_min_ in histogram analysis is an independent factor that is correlated with DFS in cervical cancer patients treated with chemo-radiation therapy.

## Background

Uterine cervical cancer is one of the most common malignancies in the female gynecologic system. Due to extremely unbalanced economical and healthcare levels across the world, the incidence and prognosis of cervical cancer vary dramatically among different countries and areas. Most cervical cancer patients in China are at advanced stages when diagnosed. According to the FIGO stage system and NCCN guidelines, locally advanced cervical carcinoma (LACC) usually refers to stage IB2 to IVA cancers, and concurrent chemoradiotherapy (CCRT) is suggested as the standard treatment strategy for such patients [1]. The current concept of cancer treatment calls for personalized treatment in order for the patient to benefit the most and to maximally increase survival time.

Among the factors that are related to the survival rate of cervical cancer, tumor size is the most well-accepted one; it has already been included as a key factor in the FIGO staging system. Other risk factors include positive lymph nodes, parametrial extension, positive surgical margins, lymph vascular space invasion, and depth of invasion [2], but most of this information can only be acquired by operational pathology, which is impossible for LACC patients subjected to radical radiation. Imaging has the ability to provide more information in vivo before treatment, so much research has been carried out on the selection of imaging parameters, such as enhancing patterns, to predict the survival rate of cervical cancer. Comparing with dynamic contrast enhanced parameters, DWI has obvious advantages, such as nonuse of contrast radium, relatively stable image quality, and a universally accepted quantitative index, the apparent diffusion coefficient (ADC).

The results regarding ADC values in predicting treatment effects vary greatly. Some studies showed low ADC values are related with recurrence and a poor survival rate [3, 4], while some found low ADC values in patients with good treatment responses [5, 6]. Other studies concluded that the available evidence is insufficient to use pre-treatment ADC values to predict treatment efficacy [7–9]. One of the main disadvantages of ADC measurements is that the location and the sizes of regions of interests (ROIs) may affect the values, which is a common practical problem in almost all parameter measurements. Therefore, the application of histogram analysis (HA) is validated, which generates more indices to describe the distribution of each voxel within ROIs, instead of a single mean value. Studies on HA did achieve inspiring results in predicting cervical cancer survival rates, but large differences exist between studies. One of the possible reasons may still be the choice of ROIs, as some studies used maximum single slices instead of whole tumor slices. We suspected that whole lesion ROIs are able to reflect lesion characteristics to the greatest extent, while small ROIs or single slice ROIs all have the possibility of selection bias. Therefore, in this study, we use whole lesion ROIs and HA to assess the possibility to use ADC values to predict disease-free survival (DFS) of cervical cancer for a follow-up period of 5 years.

## Methods

### Patients

Informed consents were waived by the institutional Ethics Committee for this retrospective study. All patients with pathologically diagnosed squamous cell uterine cervical cancer in our institute from 2009 to 2013 were reviewed, and 152 consecutive patients were found to meet the following inclusion criteria: (a) the tumor was in stage IB to IVA, (b) the patient received concurrent chemoradiation in our radiation department, (c) pre-treatment pelvic MR was performed in our hospital and was within 30 days before treatment started, and (d) valid follow-up information could be acquired from the medical records. After retrieving MR images and clinical medical records, 49 patients were excluded, including 32 with sagittal instead of axial DWI, 8 with poor quality DWI images, 5 with a tumor with a long diameter of less than 1 cm, 1 with small cell lung cancer, and 3 with a follow-up time of less than 2 years. We recorded the relevant clinical information, including age, grade, FIGO stage, and serum levels of squamous cell carcinoma antigen (SCC-Ag). In total, 103 subjects were enrolled in the study. The follow-up time was defined as from the date of first radiation to either the recurrence date or the last visit date with no events. The median follow-up time for the whole cohort was 48.9 months (range 2.0–94.6 months). Among them, 55 patients free of recurrence or metastasis at the end of the follow-up period (median follow-up time 59.7 months).

### Treatment and outcome evaluation

All patients underwent standard radiotherapy in combination with concurrent cisplatin-containing chemotherapy. Radiotherapy consisted of external beam radiation (40–50.4Gy in 1.8-2Gy daily fractions using 10-MV photons) and intracavitary brachytherapy (30–35 Gy to point A by 5 Gy fractions in 3 weeks). Six courses of cisplatin-based chemotherapy (40 mg/m^2^ dosage) were delivered weekly at the same time with radiotherapy.

The primary outcome was DFS, defined as the period of time from the first radiation treatment to the date of developing any recurrence (local or distant relapse, metastasis). Patients with persistent disease were considered to have relapsed on the first day of completing radiation therapy. Recurrence data were taken from the medical records, with evidence based on the diagnosis by the treating physician and imaging (18F-fluorodeoxyglucose [18F-FDG] positron emission tomography/computed tomography [PET/CT], MRI, CT) or biopsy results. Recurrence group and non-recurrence group were divided based on DFS ≤ 2 year and DFS > 2 years.

### MR imaging

All examinations were performed on 1.5-T MR scanners (GE optima 1.5 T MR360 and GE signa 1.5 T EchoSpeed Plus) with four-channel or eight-channel torso phased-array body coil. The scanning range was set to cover the entire pelvis from the level of the anterior superior iliac spine to the inferior level of the symphysis pubis. All patients were required to fast at least 2 h before the examination. After excluding the contradictions, patients were asked to inject 5 ml of anisodamine about 20–30 min before the examination to reduce bowel motion artifacts.

MR sequences included T1WI, T2WI, and DWI. The standard MR scan protocol was kept identical each time and was as follows: (a) axial T1WI: fast spin-echo (FSE) sequence, repetition time (TR) = 726 ms, echo time (TE) = 12 ms, matrix size = 288 × 256, field of view (FOV) = 26–36 cm, slice thickness/intersection gap = 8/1 mm, number of excitations (NEX) = 1; (b) axial T2WI: FSE-XL, TR = 5182 ms, TE = 80 ms, matrix size = 352 × 320, FOV = 26–30 cm, slice thickness/intersection gap = 8/1 mm, NEX = 2; (c) sagittal T2WI: FSE-XL, TR = 4138 ms, TE = 102 ms, matrix size = 320 × 288, FOV = 24–36 cm, slice thickness/intersection gap = 5/1 mm, NEX = 4; (d) axial DWI: single-shot echo-planar imaging sequence, TR = 3500 ms, TE = minimum time, matrix size = 128 × 128, FOV = 36 cm, slice thickness/intersection gap = 8/1 mm, NEX = 4, b-value = 0 and 1000 s/mm^2^. All slice lines of DWI were copied from the axial T2WI to make sure the images were all at the same table position.

### Image analysis

**Fig. 1:**
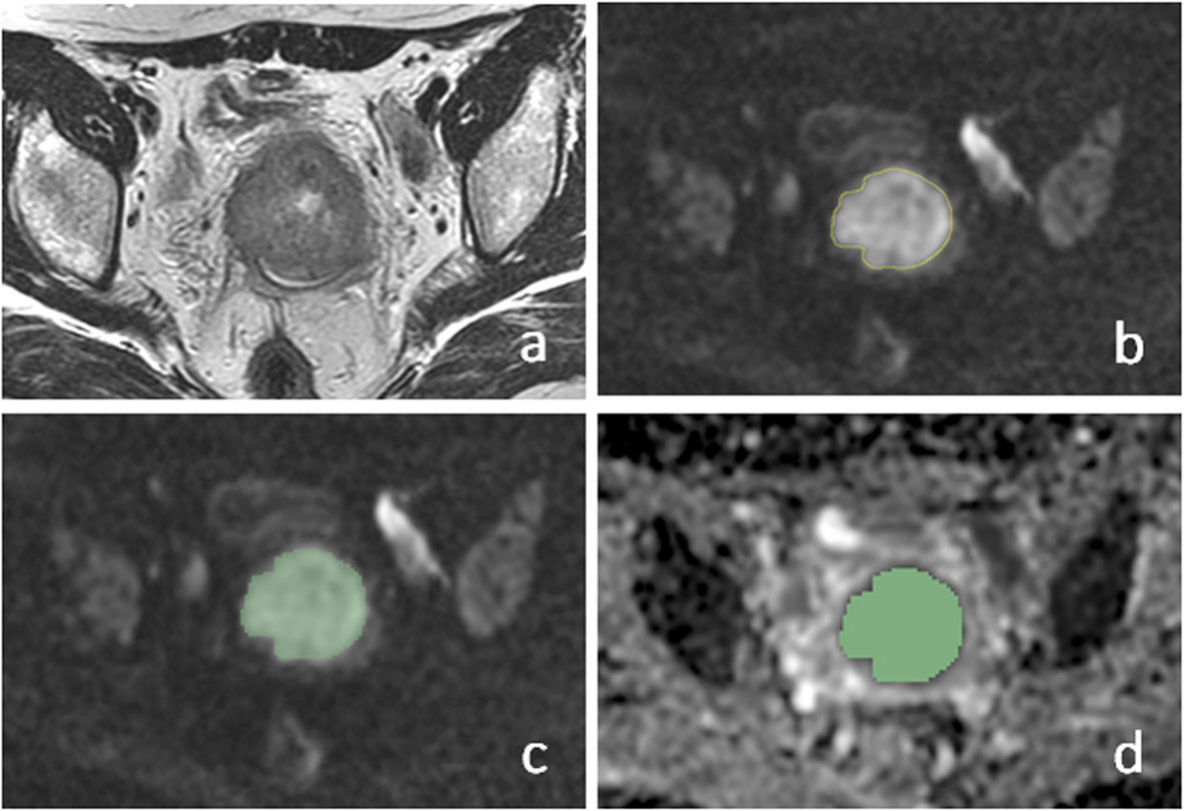
Manual segmentation of ROIs in cervical lesion and schematic diagram of parameters. **a**–**c** Referring to T2WI and DWI, ROIs were drawn manually slice by slice on DWI images along the edge of the lesions in order to cover as much tumor area as possible without excluding cystic or necrotic areas. **d** The same ROIs were registered to ADC maps

All images were retrieved from local PACS (Huahai, China). By putting axial T2WI and DWI images side by side and also referring to the sagittal T2WI, ROIs were drawn manually slice by slice on DWI images along the edge of the lesions in order to cover as much tumor area as possible without excluding cystic, hemorrhagic or necrotic areas (Fig. 1). ROIs were initially drawn by a junior radiology resident (Z.B.) with 3 years’ experience in MR reading and then reviewed and corrected by a senior attending radiologist (C.K.) with more than 10 years’ experience in gynecologic imaging. Both radiologists were blinded to patients’ outcomes during the data collection period. Whole lesion ROIs were thus achieved to acquire volumetric data of tumors.

Axial DWI images were loaded into a post-processing workstation (GE AW 4.6) and parametric ADC maps were generated automatically in the FUNCTOOL program. Parametric maps along with DWI and ROIs were all transferred to house-made radiomics software based on the 3D Slicer platform. ROIs were registered to the parametric maps. Histograms and corresponding ADC parameters values were automatically generated by the software. Excel software was used to sum up the parameters values of ROI at all slices of the tumor respectively and then calculate the average value. The HA program in the software was generated the following 8 parameters for ADC values: mean, median, maximum, minimum, 10th percentile, 90th percentile, kurtosis, and skewness. The maximal diameter of tumor (MDT) was calculated on two-dimensional axial ADC metrics.

### Statistical analyses

Continuous variables are presented as mean ± SD. Categorical variables are presented as counts and percentages. The clinical variables and ADC parameter values were compared between the recurrence and non-recurrence groups using the Wilcoxon rank-sum test or the chi-square test. Univariate and multivariate Cox regression analyses were used to evaluate the potential prognostic value of ADC parameters and relevant clinical variables (i.e., age, MDT, grade, FIGO stage, SCC-Ag) for DFS. For the multivariate analysis, parameters were selected by using stepwise selection and by considering the following covariates with a *P*-value less than 0.3 in the univariate analysis. The receiver operating characteristic (ROC) curve was drawn to determine the cutoff value of the parameters using the maximum Youden index. The Kaplan–Meier survival curve was drawn and log-rank estimates were obtained. All statistical analyses were performed using SPSS 22.0. All reported values are two-tailed, and *P*-values < 0.05 were considered statistically significant.

## Results

Clinical characteristics are listed in Table 1. All patients were followed up for more than 2 years, the median follow-up period was 59.7 months (range, 25.5–94.6 months) for surviving patients. Patient characteristics are listed in Table 1. Of the 103 women analyzed, tumor recurrence was observed in 29 cases (28.2%) at the end of the follow-up period; 11 in the first year, 9 in the second year, and 9 patients suffered from recurrence after more than 2 years of follow-up. Of the 29 patients with tumor recurrence, 3 had persistent cervical cancer, 15 patients showed distant metastasis, and 11 patients showed local relapse in the pelvic cavity. The 1-year and 2-year DFS rates were 89.3 and 80.5%, respectively. Patients characteristics in the recurrence and non-recurrence groups are presented in Table 2. Pre-treatment ADC_min_ and ADC_10%_ were 0.588 (0.163–5.351) (× 10^− 3^ mm^2^/s) and 0.776 (0.543–8.134) (× 10^− 3^ mm^2^/s) in the recurrence and non-recurrence groups, respectively.

**Table 1:** Patient and Tumor characteristics

Characteristic	Value
Patient number	103
Age (years)^a^	51 (27–78)
Histology	Squamous cell carcinoma
FIGO stage	
IB	18 (17.5%)
IIA/B	16/48 (15.5%/46.6%)
IIIA/B	6/13 (5.9%/12.6%)
IV	2 (1.9%)
Grade of differentiation	
Well/moderate	59 (57.3%)
Poor	44 (42.7%)
MDT (mm)	45.1 (14.8–90.6)
SCC-Ag (ng/ml)	4.0 (0.4–81.9)
Follow-up time (months)	48.9 (2.0–94.6)

Note: Data are number and data in parentheses are percentages; ^a^Data are median and data in parentheses are rang; *FIGO* International Federation of Gynecology and Obstetrics, *MDT* Maximal diameter of the tumor, *SCC-Ag* Squamous cell carcinoma antigen

**Table 2:** Characteristics of patients and values of ADC histogram parameters in recurrence (DFS ≤ 2 years) and non-recurrence (DFS > 2 years) groups

Parameter	Non-recurrence *n* = 83 (80.6%)	Recurrence *n* = 20 (19.4%)	*P*-value
Age (years)	50.4 ± 9.2	51.3 ± 11.4	0.973
Grade			
Well/moderate	48 (46.6%)	11(10.7%)	0.075
Poor	35 (34.0%)	9(8.7%)	
MTD	42.9 ± 16.0	48.4 ± 13.1	0.115
FIGO stage			0.996
IB/IIA	29(28.2%)	7(6.8%)	
IIB/III/IV	54 (52.4%)	13(12.6%)	
SCC-Ag	9.7 ± 14.4	11.3 ± 15.2	0.957
Median (×10^−3^ mm^2^/s)	1.094 ± 1.117	0.912 ± 0.537	0.443
Max (× 10^− 3^ mm^2^/s)	2.202 ± 1.664	2.144 ± 0.328	0.352
Mean (×10^−3^ mm^2^/s)	1.056 ± 0.156	1.013 ± 0.119	0.372
Min (×10^−3^ mm^2^/s)	0.648 ± 0.570	0.356 ± 0.383	0.001*
10% (×10^−3^ mm^2^/s)	0.900 ± 0.817	0.757 ± 0.173	0.048*
90% (×10^−3^ mm^2^/s)	1.437 ± 1.470	1.305 ± 0.219	0.900
Kurtosis	5.575 ± .254	4.910 ± 2.541	0.286
Skewness	1.184 ± 0.602	0.912 ± 0.537	0.067

Note: Data are presented as mean ± SD, with Wilcoxon rank-sum test. Data are presented number (percentages), with chi-square test. *P-value < 0.05

The values of ADC_min_ (*P* = 0.001) and ADC_10%_ (*P* = 0.048) were significantly lower in the recurrence group than in the non-recurrence group. In the univariate analysis, age, ADC_min_, ADC_10%_, and ADC_skewness_ were associated with DFS (Table 3). In the multivariate Cox analysis, ADC_min_ of the tumor was the most significant predictor for patient DFS (*P* = 0.006, HR = 0.110), where a higher ADC_min_ was significantly associated with poor DFS (Table 3).

**Table 3:** Univariate and multivariate Cox regression analysis of clinical factors and ADC histogram parameters for predicting disease-free survival

Parameter	Univariate analysis	*P*-value	Multivariate analysis	*P*-value
Age (years)	1.026 (0.987–1.066)	0.194*	1.004 (0.963–1.046)	0.858
Grade				
Well/moderate	1.0			
Poorly	2.731 (1.242–6.005)	0.412		
MTD	1.004 (0.981–1.027)	0.764		
FIGO stage				
IB/IIA	1.0			
IIB/III/IV	1.256 (0.572–2.760)	0.570		
SCC-Ag	1.004 (0.982–1.027)	0.716		
Median (×10^−3^ mm^2^/s)	0.435 (0.037–5.075)	0.507		
Max (× 10^− 3^ mm^2^/s)	0.918 (0.613–1.373)	0.676		
Mean (×10^−3^ mm^2^/s)	0.397 (0.032–4.968)	0.474		
Min (×10^−3^ mm^2^/s)	0.171 (0.063–0.463)	0.001#	0.110 (0.023–0.538)	0.006#
10% (×10^−3^ mm^2^/s)	0.106 (0.006–1.933)	0.130*	1.803 (0.069–46.817)	0.723
90% (×10^−3^ mm^2^/s)	0.793 (0.287–2.193)	0.655		
Kurtosis	0.950 (0.821–0.099)	0.489		
Skewness	0.657 (0.350–1.233)	0.188*	0.724 (0.368–1.463)	0.350

*P < 0.3; #*P* < 0.05

Hazard ratio (HR) data are reported per one-unit increase

Data in parentheses are 95% confidence intervals

**Fig. 2:**
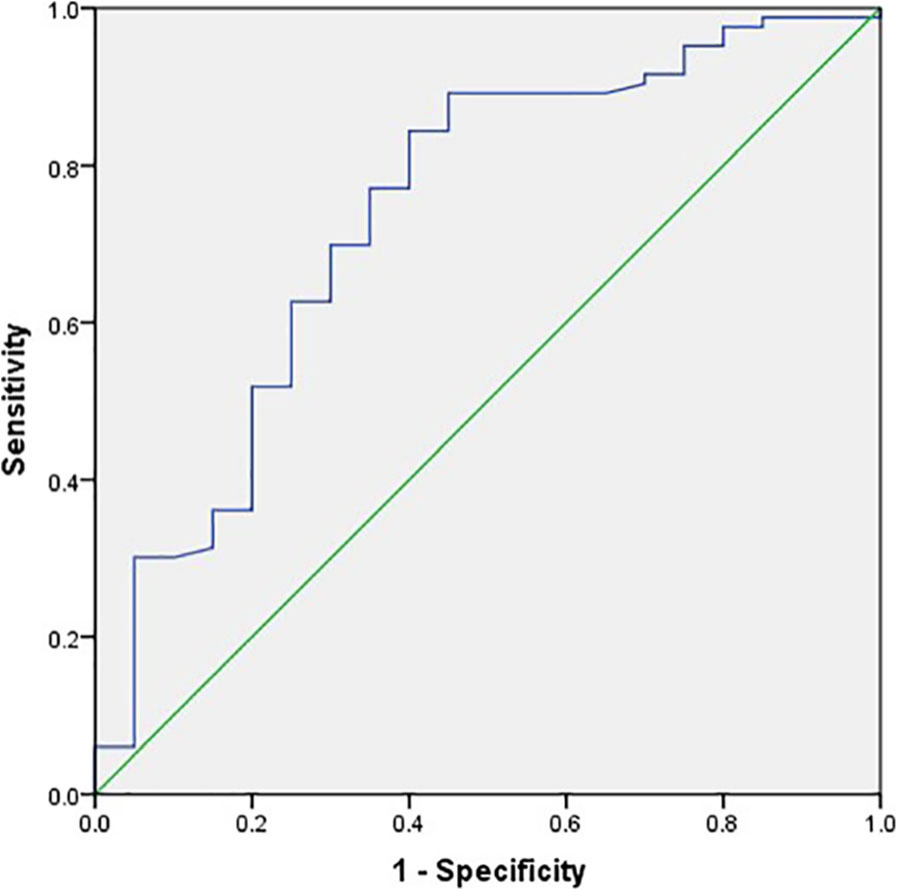
ROC curve of ADC_min_ value for predicting non-recurrence within 2 years. The cutoff value of ADC_min_ was 0.482 × 10^− 3^ mm^2^/sec, with a sensitivity of 84.3% and a specificity of 60%. The area under the ROC cure was 0.738 (95% CI, 0.608–0.868; *P* = 0.001)

**Fig. 3:**
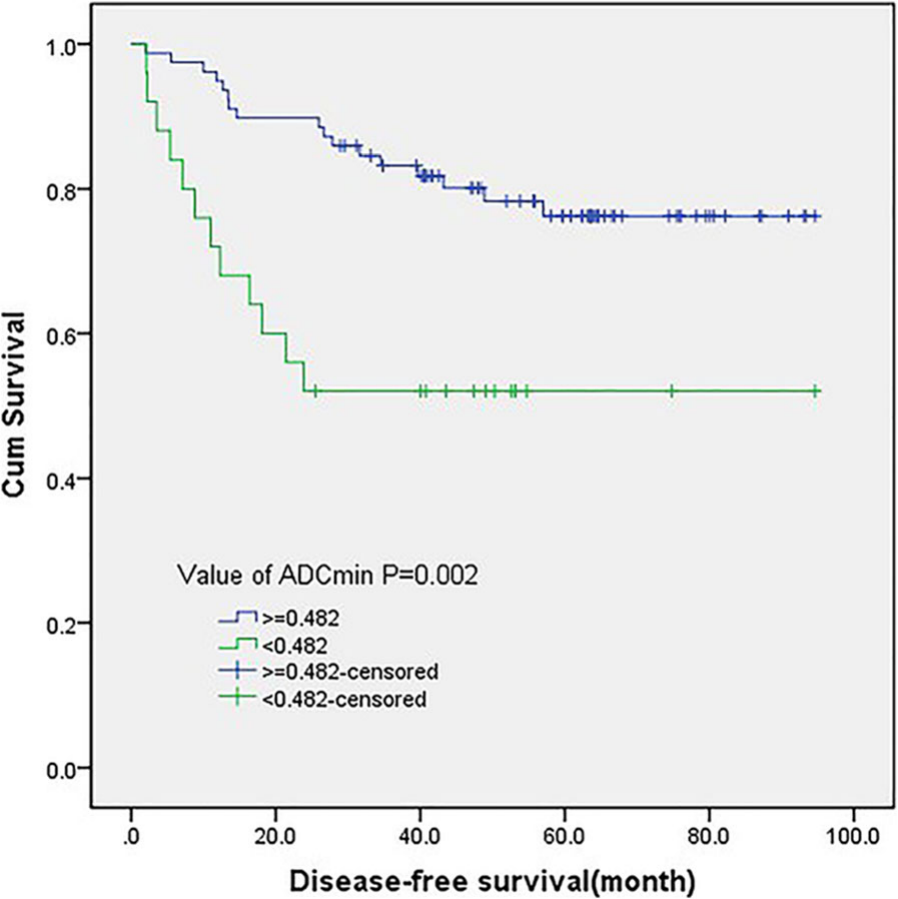
Kaplan–Meier curve for disease-free survival with ADC_min_ value. A statistically significant difference in DFS is observed between patients with ADC_min_ ≥ 0.482 and ADC_min_ < 0.482 (*P* = 0.002, long-rank test)

ROC analysis of tumor recurrence within 2 years showed that the area under the curve for ADC_min_ was 0.738 (95% CI, 0.608–0.868; *P* = 0.001) (Fig. 2). The cutoff value of ADC_min_ was 0.482 × 10^− 3^ mm^2^/sec, with a sensitivity of 84.3% and a specificity of 60%. The DFS for patients with ADC_min_ ≥ 0.482 mm^2^/sec was significantly higher than that of patients with ADC_min_ < 0.482 mm^2^/sec (*P* = 0.002, long-rank test) (Fig. 3).

## Discussion

Although CCRT is an optimal therapy for LACC with appreciable outcome, treatment for relapse of tumor afterwards remains tough. Thus we assumed it will be of clinical significance to find high-risk patients who subject to recurrence within short time, and who might benefit from additional or novel therapies, such as targeted agents with chemotherapy [10], or adjuvant or consolidation chemotherapy after CCRT [11]. Therefore, functional MR sequences and imaging processing techniques been investigated for this purpose.

In our study, ADC_min_ and ADC_10%_ were significantly different between the recurrence and non-recurrence groups after 2 years of follow-up, while only ADC_min_ was found to be correlated with DFS of cervical cancers by multivariate regression analysis. ADC_10%_ and skewness were not associated with tumor recurrence in the multivariate analysis.

In previous studies, ADC values were found to be lower in cervical squamous cell carcinoma compared with adenocarcinoma, even when using HA-derived mean ADCs [3, 12] and minimal ADCs [13, 14]. Therefore, we only included squamous cell cancers in this study, as it is the most common histologic type of cervical cancer and also to exclude possible interference due to different pathology types. ADC values of malignant tumors are commonly lower than those of normal tissues, as is also the case in uterine cervical cancer [3, 15]. However, the usefulness of mean ADC values in predicting therapeutic effects differs between studies, possibly due to inter-observer variations in ROI selection and size. The introduction of HA, by further dissecting and defining the distributions of all voxels involved, helps to provide more information for tumor characterization. Theoretically, only ROIs that cover whole lesions are able to comprehensively reflect tumor characteristics. Even necrosis may represent one of the intrinsic features of a tumor, so we think it is not necessary to exclude the possible necrotic areas, as was usually done in previous studies when using small ROIs.

With the method of HA, lower ADC values were often found to be related to more aggressive and poor prognosis tumors, such as poorly differentiated stage I cervical cancers, compared with well/moderately differentiated ones [16]. In a study by Erbay et al. [17], using HA to achieve several percentile ADC values, they found ADC_50%_, ADC_75%_, ADC_90%_, and ADC_95%_ were lower in patients with tumor recurrence than in those without recurrence. Unfortunately, the percentiles they used were all above 50%. Similar results were also found in a study by Gladwish et al., in which, among different percentiles of ADC values that were higher than 50%, the ADC_95%_ and nADC_95%_ (normalized to urine) were found to be independent protective factors associated with DFS [18]. Another study showed ADC_75%_ to be a risk factor for tumor recurrence [19]. In our study, high percentiles do not seem to be so significant among all histogram parameters, while the lower ADC values (ADC_min_ and ADC_10%_) exhibit a difference between the recurrence and non-recurrence groups. Several studies have also shown that ADC_10%_ is significantly associated with event-free survival [20] and DFS [21]. However, in our study, low ADC_10%_ values may be correlated with recurrence within 2 years, yet they are not associated with DFS in our Cox regression analysis; ADC_min_ is an independent risk factor related to DFS using Cox regression analysis.

Some studies also found that ADC_min_ is a valuable factor that reflects tumor aggressiveness. The ADC_min_ of stage T2b-T4 cervical cancers was significantly lower than that of stage T1 to T2a cancers, and it was even lower in patients with lymph node metastasis or distant metastasis [22]. In one histopathologic study, the ADC_min_ value of cervical cancer exhibited a negative relationship with Ki-67, the index of cellular proliferation (r = − 0.56, CI = − 0.68−− 0.43, [23]). A meta-analysis among various kinds of tumors also found ADC_min_ had a stronger relationship with Ki-67 compared with ADC_mean_ [24]. In a study on malignant astrocytomas, ADC_min_ was found to be negatively correlated with the Ki-67 labeling index, which indicates poor prognosis, and the ADC_min_ value was found to be significantly higher in the stable group than in the progressing group after treatment [25]. Such results confirm the possible application of ADC_min_ in predicting survival rates; higher ADC_min_ values may be related to longer DFS, as is also shown in the present study. However, contradictions can also be found in the literature. For example, in a study by Marconi et al. [26], ADC_min_ was found to be an independent factor related with DFS as well, but it was a risk factor instead of a protective factor. Considering the fact that HA can also be affected by the coverage extent of ROIs, studies with whole tumor ROIs or single slice ROIs may lead to different results. As stated above, we assumed that whole lesion ROIs can reflect intrinsic tumor heterogeneity to the highest extent, so they are more reliable. The different b-values used for DWI scanning form another factor that may affect the results, because there is no single b-value that is uniformly used among studies.

## Conclusions

The volumetric ADC_min_ in HA has the possibility to act as an independent risk factor that is correlated with DFS in cervical cancer patients treated with chemo-radiation therapy. Pre-treatment ADC histogram analysis may identify higher risk of recurrence patients, which will enable greater personalization of cancer care either via a priori treatment decisions or subsequent follow-up regimens.

## Data Availability

The datasets used and analyzed during the current study are available from the corresponding author on reasonable request.

## Abbreviations

ADC: Apparent diffusion coefficient
CCRT: Concurrent chemoradiotherapy
DWI: Diffusion weighted images
FIGO: International Federation of Gynecology and Obstetrics
HA: Histogram analysis
LACC: Locally advanced cervical carcinoma
MDT: Maximal diameter of tumor
NCCN: National Comprehensive Cancer Network
OS: Overall survival
ROI: Regions of interests
ROC: Receiver operating characteristic
